# Acute Effects of Partial Range of Motion Resistance Training and Increases in Blood Lactate Impact Accuracy of Penalty Kicks in Soccer Players

**DOI:** 10.1155/2022/4769560

**Published:** 2022-06-08

**Authors:** Mariusz Ozimek, Tadeusz Ambroży, Tatiana Krasavina, Irina Lazareva, Christina Popova, Łukasz Rydzik, Vitaly Rybakov, Konstantin Gurevich, Stefane Dias, Brian Binkley, Rokaya Mikhailenko, Alexander Tsymbal, Emilian Zadarko, Victoria Zaborova

**Affiliations:** ^1^Institute of Sports Sciences, University of Physical Education, 31-571 Krakow, Poland; ^2^Institute of Clinical Medicine, Sechenov First Moscow State Medical University (Sechenov University), 119991, Trubetskaya Street, 8/2, Moscow, Russia; ^3^Sports Adaptology Laboratory, Moscow Institute of Physics and Technology (National Research University), 141700, Institutskiy Pereulok 9, Dolgoprudniy, Moscow Region, Russia; ^4^UNESCO Chair Healthy Lifestyle for Sustainable Development, Moscow State University of Medicine and Dentistry, 127473, Delegastkaja Street, 20/1, Moscow, Russia; ^5^Department of Health and Human Performance and Exercise Science, Keiser University, 5600 Lake Underhill Rd. Orlando, Florida 32807, USA; ^6^College of Medical Sciences, Institute of Physical Culture Studies, University of Rzeszow, Poland

## Abstract

The purpose of this investigation was to assess the acute effects of partial range of motion (pROM) exercises, on the accuracy of soccer penalty kicks on goal. This method limits the joint from moving through the complete length of a motion, creates an occlusion effect, and thus causes the type 1 muscle fibers to work anaerobically. Thirty-six soccer players, with 5-8 years of soccer playing experience, were pretested for accuracy then retested (rtt = 0.92) and divided into random groups from the Associação Banco do Brasil Futebol Clube—Group A, Paraná Futebol Clube—Group P, and Coritiba Futebol Clube—Group C. Groups were composed of 12 people performing full range of motion (fROM) exercises or pROM exercises. Both groups performed 5 sets of back squats at 50% of body weight in sets of 40 seconds with metronome tempo of 56 bpm for an average of 10-12 repetitions per 40-second set. Blood samples were collected post-warm-up, after the 3rd set, and following the 5th set for both groups, within 3–5 minutes of cessation of exercise. Athletes performing fROM exercises showed increased blood lactate from 2.69 ± 0.2 to 4.0 ± 1.2 mmol/L (*p* < 0.05), and in pROM, blood lactate increased from 2.48 ± 0.42 to 10.29 ± 1.3 mmol/L (*p* < 0.001). In fROM, accuracy decreased from 42.96 ± 13.39% to 41.37 ± 17.25% (*p* > 0.1), a slight decrease, while in the pROM groups, accuracy decreased from 45.42 ± 14.93% to 24.53 ± 10.2% (*p* < 0.001). The calculations demonstrating average percentages of accuracy are presented in the tables. These findings support that pROM exercises significantly increase blood lactate resulting in a reduction in soccer kick accuracy. This decrease in accuracy directly correlates to the accumulation of lactic acid and hydrogen ions (H+) and demonstrates that pROM strength training should not be utilized prior to a sport-specific session in order to avoid interference with the development of special skills.

## 1. Introduction

The training of soccer players is associated with vast amounts of general, special physical, and sport-specific training, to develop mastery of necessary technical skills [1–7]. Strength development is one of the most important physical characteristics to cultivate in soccer players [8, 9]. Therefore, it is necessary to determine the best means and methods of strength training, then arrange them in such a way that they do not have a negative impact on sport performance during competition and practice. Previous research has shown that a high level of speed-strength preparedness is crucial when performing agility movements like sudden direction change, sprints, accelerations, and jumps during matches [10–12]. It is also known that strength training leads to a change in the properties of the neuromuscular apparatus, which could interfere with the effectiveness of the implementation of motor skills during execution of sport-specific movements [13–15]. The bioenergetic and biomechanical demand of soccer training can significantly impact the development of both, having either a positive or negative influence upon the development of the athlete's performance mainly related to changes in the operation of sensory systems and motor pattern execution. Moreover, training is only effective for a soccer player under competitive conditions if a positive transfer of physical load can transition into accuracy of the motor actions [16, 17]. Scientific literature informs us that negative effects are most often associated with a nonspecialized nature of a load, whereas the opposite is true of highly specialized work, which contributes to positive transfer to the athlete's performance, enhancing the accuracy of both acute and subsequent skill development and execution [18].

There are many forms of strength training; among them are isometric, full range of motion (fROM), and partial range of motion (pROM). During pROM work with the intensity of muscle tension within 30%-60% of the athlete's 1 rep max (1RM), vascular occlusion begins, which leads to the activation of anaerobic glycolysis and is felt by the athlete as a “burning sensation” in the working muscles as hydrogen ions (H+) accumulate in the tissues [19, 20]. It is known that the accumulation of H+ during fatigue significantly reduce the efficiency of the performance of motor actions desired athletically for improved execution of a specific skill. Thus, fatigued muscles that have not fully recovered are not able to adequately perform specialized work [21, 22]. Strength training methods include static, dynamic, and static-dynamic exercises, although the most widely known method is the use of dynamic exercises for building muscle mass, when the intensity is 60-70% of the repeated maximum (RM), and the number of repetitions is 6-12 times [23]. When using statodynamic exercises, the intensity is 10-60% of the RM; the exercises are performed without muscle relaxation and induce a strong “pain sensation” [22]. The different nature of muscle work predetermines the characteristics of physiological processes. When performing dynamic strength exercises, the blood supply to the muscles is not disturbed. In this case, oxidative muscle fibers (OMF) do not accumulate lactic acid because mitochondria use lactate and hydrogen ions for oxidative phosphorylation. An increase in the concentration of hydrogen ions in muscle fibers (MV) is a factor that stimulates anabolic processes; therefore, the training effect should be mainly on glycolytic muscle fibers (GMF). When performing static-dynamic strength exercises, acidification will occur in both oxidative and glycolytic muscle fibers. The accuracy of the motor action largely depends on the state of the executive apparatus.

Unlike the pROM exercises, fROM resistance training exercises contribute to the development of strength while not having a significant impact on the performance of technical or sport-specific actions. Performing pROM exercises demands maintaining tension in the active muscles, with only a small amplitude of change in the angles of the joints [24, 25]. Therefore, it is necessary to study the problem of constructing an optimal training process that includes the methods of increasing strength without significantly distorting the abilities and skills of performing sport-specific actions in soccer competition [26]. Local strength exercises can be performed in various ways. For example, working at an intensity of 50-60% of 1RM, the duration of the strength exercise reaches 60 seconds, about 15-20 repetitions. From our point of view, during the exercise, low-threshold motor units (MUs) are first recruited, and then, as the reserves of adenosine triphosphoric acid (ATP) and creatine phosphate (CrP) are depleted, higher-threshold MUs (HMUs) are connected to work. As a result, lactic acid is not formed in OMV but is formed only in glycolytic MB.

If you perform static-dynamic exercises, exercises that do not allow complete relaxation of active muscles, then due to the cessation of blood circulation, after the depletion of the reserves of ATP and CrF, lactic acid should accumulate in the OMV, OGMV, and GMV. Therefore, it should be significantly higher compared to dynamic strength exercises.

Thus, it can be assumed that the fulfillment of dynamic local strength exercises should not lead to significant acidification, and acidification does not occur at all in OMP; therefore, there should not be a decrease in precision motor actions, for example, strikes on a goal at a target. The implementation of static-dynamic local strength exercises should lead to acidification of the OMV; therefore, the accuracy of strikes on the goal within 10-15 minutes after strength exercise should be reduced. The decrease in accuracy will be associated with a discrepancy between the muscle control program and the state of the executive apparatus.

One of the primary sport-specific actions in soccer is penalty and free kicks, and the outcomes of these technical actions can significantly affect the outcome of the game. Tests aimed at determining accuracy of kicks on the target in soccer are greatly informative [27]. The purpose of this study was to investigate the acute effects of fROM and pROM resistance training exercises on the accuracy of kicks in soccer players. We hypothesized that the accuracy of kicks would be reduced with the use of pROM resistance training exercises, and accuracy correlates to blood lactate levels.

## 2. Materials and Methods

### 2.1. Experimental Approach to the Problem

The study involved 36 players that were pretested for their accuracy (44 ± 10%) of penalty kicks and retested (test-retest reliability, trr = 0.92) then placed into three stratified random groups from the Associação Banco do Brasil Futebol Clube (indoor soccer)—Group A; Paraná Futebol Clube (soccer)—Group P, and Coritiba Futebol Clube (soccer)—Group C. Each group was composed of 12 players (A = 12, P = 12, and C = 12), which were further subdivided into 2 groups of 6 players each, one group from each team (A, P, and C) performing full range of motion exercises (fROM) and the other using partial range of motion exercises (AfROM = 6, ApROM = 6, PfROM = 6, PpROM = 6, CfROM = 6, and CpROM = 6). Thus, there was a 2 : 1 ratio between the type of soccer played by the participants, 24 soccer players and 12 indoor soccer players in total. To test the accuracy of penalty kicks, the players performed 25 shots, divided in to 5 sets of 5 kicks at the target. The indoor soccer team target was placed at a distance of 9 meters, and the outdoor soccer team at a distance of 11 meters. After executing each set of 5 penalty kicks, there was a passive rest interval of 4 minutes during which the percentage of shots on target was calculated. Once all the sets were completed, the total number of kicks that successfully hit the target was calculated to determine the overall accuracy with alpha level significance (*p* ≤ 0.05). After a one-week period of familiarization, all the players were divided into pairs, one person performing pROM and the other fROM. Both players performed 5 sets of back squats with a barbell load corresponding to 50% of the body weight of the athlete for 40 seconds following a metronome tempo of 56 bpm with the assistance of two spotters to place and remove barbell from athletes' back per set. Each repetition of squat exercise was performed with a change in joint angle and body position correlating with the eccentric phase for 2 beats then concentric phase for 2 beats totaling 4 metronome beats per rep so that on average, 10-12 repetitions were performed per set.

### 2.2. Subjects

The study was conducted in Curitiba, Brazil, with the cooperation of 3 different soccer teams at their regular training facilities during the preparatory period. Groups A, P, and C had 12 people each that were further subdivided into 2 groups of 6 individuals each. This study was approved by the ethics committee of Keiser University Orlando, Protocol No. 3 on 11.12.2019. All subjects included in this study were volunteers and had parental or guardian signed consent before participation, according to the Institutional Review Board protocol, in accordance with the principles of the Declaration of Helsinki.

### 2.3. Blood Lactate Concentration Measurements

Blood sampling was taken from the index finger of each of the players, and the concentration of lactic acid was measured using the Accutrend (Roche Diagnostic, Basel, Switzerland) device three times during the study for both groups immediately following the warm-up, the 3rd set, and 5th set. All measurements were performed by the same tester. The validity of the Accutrend analyzer was guaranteed through use according to the manufacturer's instructions to measure the test samples against the lactate standards found therein [28, 29].

### 2.4. Evaluation of the Accuracy of Penalty Kicks

The players carried out penalty kicks on a 1 m^2^ target specially designed for this experiment. The target was placed on the upper, left side of the goal. In order to assess the accuracy of shots taken on the goal, the test was performed as follows: the players performed 25 shots, divided in to 5 sets of 5 kicks, at the target from a distance of 9 meters in the case of the indoor soccer team and at a distance of 11 meters for the outdoor soccer teams. After first set of 5 penalty kicks was executed, there was a passive rest interval of 4 minutes, then again after each set, and the percentage of shots to the target was calculated. After all the tests were completed, the total number of shots that successfully hit the target was calculated to determine the players overall accuracy. Prior to testing, a 15-minute warm-up was conducted by a coach who led players through a series of mobility exercises consisting of 5 minutes in a slow run, 5 minutes juggling with the ball, and 5 minutes of dynamic stretching. After a one-week period of familiarization with the exercises being used in the study, all the players were divided into pairs, one person performing pROM and the other fROM.

### 2.5. Statistical Analysis

The experimental data obtained during the research were statistically processed. The following methods of mathematical statistics were used: the arithmetic mean, standard deviation, coefficient of variation, and measurement errors of these parameters, as well as asymmetry and kurtosis were calculated. To determine the significance of the difference between the arithmetic means, the Student *t*-test for dependent or independent samples or one-way analysis of variance was used (ANOVA). The standard software package from the Excel and STATGRAPH statistical software package was used. Players in the pROM group differed from the fROM group in that the angle of flexion in the knee joint was varied from a full squat up to 100-110 degrees (avoiding full-leg extension), defining this training as partial range of motion exercises. To control the angle of the joint, video was filmed and processed using the Dartfish program.

The prescribed load on the bar and the time and pace of the squats performed were consistent for every participant. Soccer kick accuracy was assessed immediately after each set of barbell back squat exercises. Materials and Methods should contain sufficient detail so that all procedures can be repeated. It may be divided into headed subsections if several methods are described.

## 3. Results and Discussion

Eligibility criteria included 5-8 years of soccer experience, current soccer position playing midfielder and forward only, and needed to have played on their respective team for 8 months or more. Three groups of players aged 14.5 ± 0.5 years, with body weight 62.9 ± 6.6 kg and height ranging from 170 ± 5.9 cm, were involved (Table 1).

Methodological parameters of the methods used for assessing the accuracy of penalty kicks showed that the average accuracy of impacts on the 1 m^2^ meter target was 44% with an error of ±10%. Comparison of accuracy was performed by comparing the results of the 1st set of the player to the subsequent sets. The correlation between the 1st and 2nd sets wastest/retest (trr) = 0.92 showing that the tests chosen to assess the accuracy of penalty kicks on the goal are highly reliable and logically informative.

### 3.1. The Acute Influence of fROM Resistance Training on the Accuracy of Penalty Kicks

All groups performed their penalty kicks on the dominant leg and were given a 4 m running advance. Group A, fROM carried out penalty kicks, from 9 m (indoor soccer). Penalty kick accuracy before resistance training was 57.33 ± 12.56%, concentration of lactate (La) = 2.98 ± 0.4 mmol·L. After resistance training, the change in accuracy was as follows: 1st set = 50.0%; 2nd set = 46.6% (*p* < 0.05); 3rd set = 46.6% (*p* < 0.05), La = 3.98 ± 0.2 mmol·L (*p* < 0.05); 4th set = 56.6% (*p* > 0.05); 5th set = 40.0% (*p* < 0.05), La = 4.55 ± 0.4 mmol·L (*p* < 0.05); the total accuracy was 48.0 ± 7.15% (*p* < 0.1). Accuracy of penalty kicks began to decrease immediately after the 1st set, and after the 4th set returned to the initial level. At the end of the 5th set, accuracy decreased again by 30% in comparison to the trial performed without resistance training. The concentration of lactate was significantly increased in the 3rd and 5th sets, exceeding the warm-up by 25%-35%, respectively.

Athletes performing fROM exercises showed increased blood lactate from 2.69 ± 0.2 to 4.0 ± 1.2 mmol/L (*p* < 0.05), and in pROM, blood lactate increased from 2.48 ± 0.42 to 10.29 ± 1.3 mmol/L (p <0.001). In fROM, accuracy decreased from 42.96 ± 13.39% to 41.37 ± 17.25% (*p* > 0.1), a slight decrease, while in the pROM groups, accuracy decreased from 45.42 ± 14.93% to 24.53 ± 10.2% (*p* < 0.001). The calculations demonstrating average percentages of accuracy are presented in Tables 2 and 3. These findings support that pROM exercises significantly increase blood lactate resulting in a reduction in soccer kick accuracy (Tables 2 and 3).

Group C, fROM carried out penalty kicks from 11 m. The accuracy before resistance training was 33.14 ± 8.8, La = 3.11 ± 0.3 mmol·L. After resistance training, shot accuracy was measured at the following: 1st set = 25.7% (*p* < 0.05); 2nd set = 45.7% (*p* < 0.05); 3rd set = 48.57% (*p* < 0.05), La = 3.78 ± 0.2 mmol·L (*p* > 0.05); 4th set = 31.42% (*p* > 0.05); 5th set = 37.14% (*p* > 0.05), La = 4.41 ± 0.2 mmol·L (*p* < 0.05); the total accuracy was 37.71 ± 11.74% (*p* < 0.1). Accuracy of shots increased after the 2nd and 3rd set and then returned to the original level. The lactate level increased as the sets were performed in all samples.

The P, fROM group carried out penalty kicks on the goal from 11 m. The accuracy without resistance training was 38.4 ± 14.5%, La = 1.98 ± 0.2 mmol·L. After resistance training, shot accuracy was measured at the following: 1st set = 36.0% (*p* > 0.05); 2nd set = 40.0% (*p* > 0.05); 3rd set = 32.0% (*p* > 0.05), La = 2.9 ± 0.1 mmol·L; (*p* < 0.05); 4th set = 36.0% (*p* > 0.05); 5th sets = 48.0% (*p* < 0.05), La = 3.04 ± 0.2 mmol·L (*p* < 0.05); the total accuracy was 38.4 ± 9.2% (*p* > 0.1). Shot accuracy improved only after the 5th set, while the overall accuracy did not change. The lactate level increased as the sets were performed in all samples. Thus, the performance of local fROM resistance training leads to a decrease in the accuracy of penalty kicks, even with an increase in the level of lactate in the blood of only 1-2 mmol/L.

### 3.2. The Acute Influence of pROM Resistance Training on the Accuracy of Penalty Kicks

Group A, pROM carried out shots on the goal from 9 m. Shot accuracy without resistance training was 62.66 ± 4.84%, La = 2.96 ± 0.2 mmol·L. After the resistance training, the shot accuracy was measured at the following: 1st set = 40.0% (*p* < 0.05); 2nd set = 40.0% (*p* < 0.05); 3rd set = 33.3% (*p* < 0.01), La = 10.66 ± 0.4 mmol·L (*p* < 0.001); 4th set = 43.33% (*p* < 0.05); 5th set = 23.33% (*p* < 0.001), La = 10.75 ± 0.4 mmol·L (*p* < 0.001); the total accuracy was 36.0 ± 5.65% (*p* < 0.05). Shot accuracy began to decrease, starting from the 1st set and continued through the 5th set where a maximal decrease of more than 2.5 times when compared to shot accuracy post-warm-up was reached. The concentration of lactate increased more than 3.5 times up until the 3rd set but did not change after the 5th set.

Group C, pROM carried out shots on the goal from 11 m. Accuracy without resistance training was 36.8 ± 12.45%, La = 2.32 ± 0.2 mmol·L. After the resistance training, shot accuracy was measured: 1st set = 16.0% (*p* < 0.001); 2nd set = 28.0% (*p* < 0.001); 3rd set = 20.0% (*p* < 0.001), La = 6.98 ± 0.4 mmol·L (*p* < 0.001); 4th set = 24.33% (*p* < 0.001); 5th set = 8.0% (*p* < 0.001), La = 11.28 ± 0.6 mmol·L (*p* < 0.001); the total accuracy was 19.2 ± 5.21% (*p* < 0.05). Shot accuracy was minimal in the 1st and 5th sets and 1.3-1.8 times lower than without resistance training. The concentration of lactate increased more than 3 times in the 3rd set and 4.8 times after the 5th set.

Group P, pROM carried out penalty kicks from 11 m. Accuracy without resistance load was 36.8 ± 9.54%, La = 2.16 ± 0.3 mmol·L. After the resistance training, accuracy of penalty kicks was measured at the following: 1st set = 36.0% (*p* > 0.05); 2nd set = 20.0% (*p* < 0.01); 3rd set = 16.0% (*p* < 0.001), La = 6.60 ± 0.2 mmol·L (*p* < 0.001); 4th set = 16.0% (*p* < 0.001); 5th set = 4.0% (*p* < 0.001), La = 8.84 ± 0.6 mmol·L (*p* < 0.001); the total accuracy was 18.4 ± 11.52% (*p* < 0.05), which statistically shows significant poor quality of kicks. The concentration of lactate increased 3 times in the 3rd set and >4 times after the 5th set. It was observed that shot accuracy decreased as the pROM exercises were performed and the accumulation of lactic acid increased in the blood.

## 4. Comparative Analysis of the Degree of Influence of fROM and pROM Resistance Training Exercises on Accuracy of Penalty Kicks

**Figure 1:**
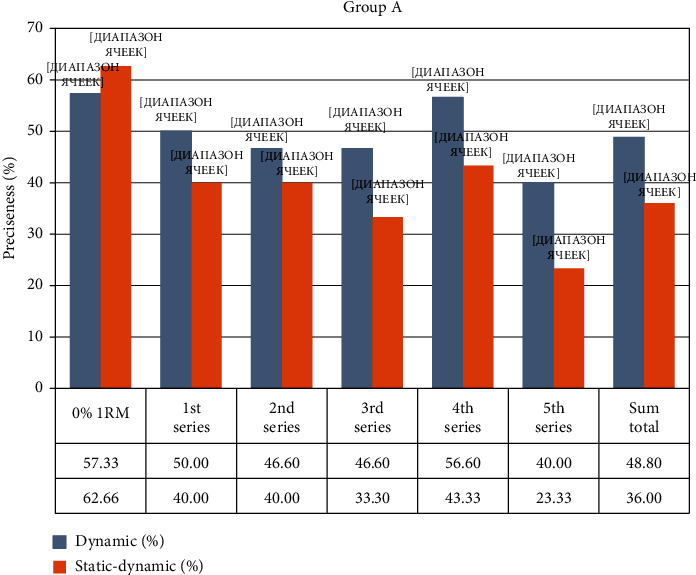
Change in accuracy during the experiment in Group A.

Figure 1 shows the effects of fROM and pROM resistance training exercises on the accuracy of shots in soccer players.

**Figure 2:**
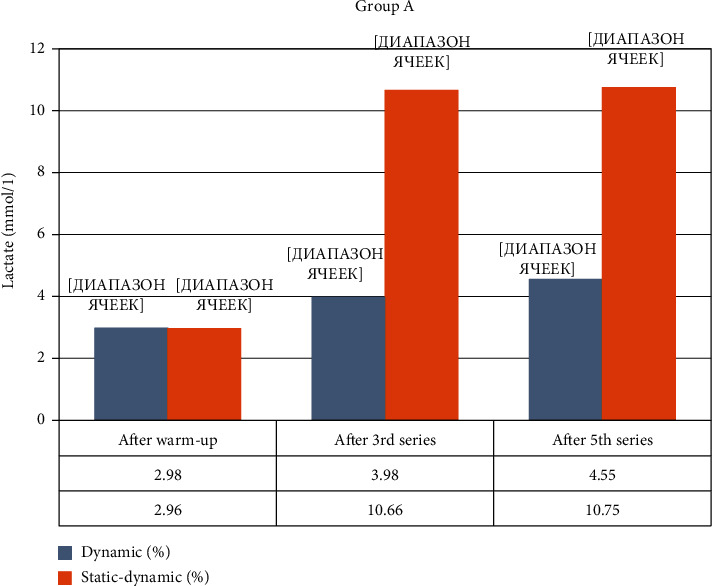
Change in lactate concentration during the experiment in Group A.

Figure 2 shows changes in the concentration of lactate in the capillary blood after performing fROM and pROM exercises.

**Figure 3:**
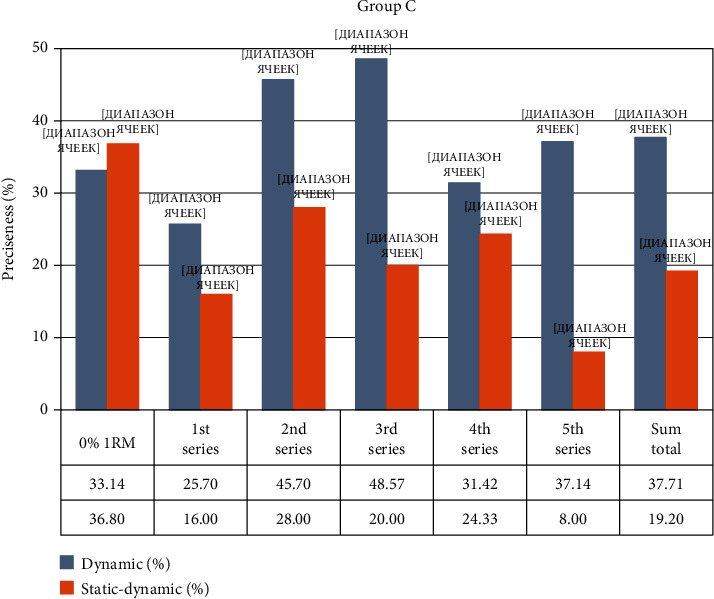
Changing accuracy during the experiment in Group C.

In Figure 3, Group C, fROM shot accuracy is significantly lower (*p* < 0.05) than that in Groups A, fROM and P, fROM after performing fROM exercises.

**Figure 4:**
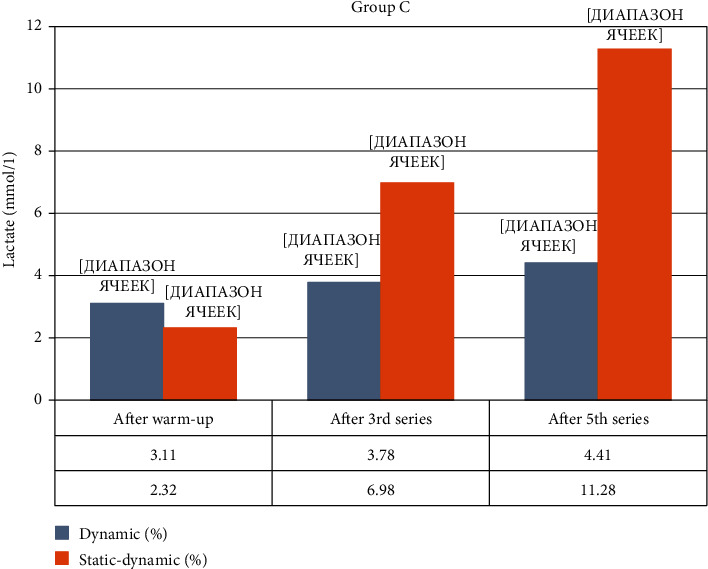
Change in lactate concentration during the experiment in Group C.

Figure 4 shows changes in lactate in the capillary blood after performing 5 sets of fROM and pROM back squat exercises.

**Figure 5:**
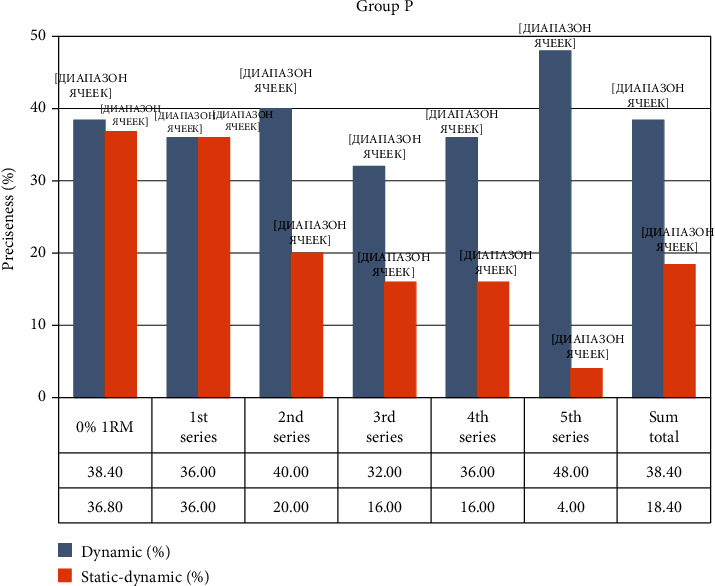
Changing accuracy during the experiment in Group P.

Figure 5 shows the data gathered on the change in shot accuracy after performing fROM and pROM resistance training exercises in Group P with P, fROM showing a tendency of increased accuracy in the 5th set.

**Figure 6:**
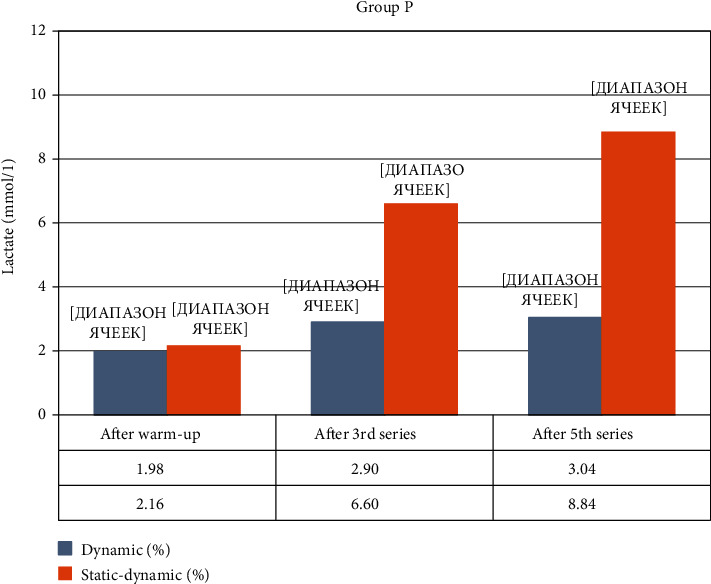
Change in lactate concentration during the experiment in Group P.

Figure 6 shows the changes in lactate concentration of capillary blood in Group P.

### 4.1. Discussion

Analysis of literary sources showed that the main factors determining the accuracy of motor actions, in particular, shots on goal in football players, are the state of the musculoskeletal system and the compliance of the motor program with it; changes in the state of muscles affect the accuracy of motor actions, both in the urgent and in the long-term aspect; however, the regularities of the relationship between strength exercises of a dynamic and statodynamic nature with the accuracy of performing strikes in football have not been studied enough [30].

The study of the acute, adaptive reaction of football players after the performance of pROM and fROM strength exercises and the influence on precision motor actions (shots on goal from 11 m) revealed the following regularities:

1. The use of dynamic strength exercises significantly increases the concentration of lactate in the blood within 2-3 mmol/L, but the accuracy of strikes may also increase slightly from 36% to 48%
2. The use of static-dynamic strength exercises leads to significant acidification of muscles and blood in the range of 8-12 mmol/L, significant local fatigue, and, as a consequence, a sharp decrease in the accuracy of hitting the target by 5-10 times

It is clear that there is an indirect correlation between the accumulation in lactate concentration in the blood and a reduction in the accuracy during execution of penalty kicks on the target [31]. In the present study, following a comparison of acute effects of pROM vs. fROM resistance training and the impact on accuracy of penalty kicks in soccer players, it was concluded that shot accuracy was negatively impacted due to the concentration of lactate in the blood of working muscles that was significantly increased by the specific nature of the execution of resistance training exercise and not on the intensity (% 1RM) of the barbell back squat. Athletes performing fROM exercises showed increased blood lactate from 2.69 ± 0.2 to 4.0 ± 1.2 mmol·L (*p* < 0.05) with a decrease in accuracy from 42.96 ± 13.39% to 41.37 ± 17.25% (*p* > 0.1). Despite the increase in acidosis in the muscles, fROM resistance training exercises did not significantly affect motor skill patterns and the performance of the muscles. It is seen that the implementation of pROM exercises leads to the accumulation of lactate in the blood that is more than 2 times that of fROM exercises when compared. With the performance of fROM exercises, there is a tendency for overall increase in accuracy.

During the study, athletes performing pROM exercises showed increased blood lactate from 2.48 ± 0.42 to 10.29 ± 1.3 mmol·L (*p* < 0.001) with a decrease in accuracy from 45.42 ± 14.93% to 24.53 ± 10.2% (*p* < 0.001). In the case of performing pROM exercises, especially when they were repeated and without achieving full recovery, accumulation of lactate and hydrogen ions and, consequently, a significant decrease in accuracy on the target were observed. In Group A (combination of A, pROM and A, fROM participants), there were statistically significant (*p* < 0.05) differences in accuracy observed after the 1st set of barbell back squats, and the maximum differences were noted after the 5th set. In the P, fROM group, we see an increase in accuracy after the 5th set, whereas the opposite tendency is observed in P, pROM as there is a systematic decrease in accuracy (after the 5th set the accuracy of the penalty kicks decreased 8.5 times). The total accuracy after performing the 5th set of pROM exercises dropped almost 3 times. When performing pROM exercises, accuracy after the 1st set decreased by 50% and after the 5th set had decreased by more than three times. This means that the pattern found in Group A, pROM was confirmed in testing results from Group C, pROM. After the fROM exercises, the concentration of lactate changes very little. However, after performing pROM exercises, the concentration of lactate increases with each set. It is seen that the concentration of lactate after fROM strength exercises increases insignificantly (*p* > 0.05) while after pROM exercises, the concentration of lactate increases significantly (*p* < 0.05) and reaches its maximum values by the fifth set (11.28 mmol/L).

This study showed that performing pROM exercises with an intensity of the load corresponding to 50% of the athlete's body weight leads to greater increase in the concentration of lactate than when performing fROM exercises at the same intensity. These results agree with the results of research performed by another scientific, research study group from the Institute for Biomedical Problems, Moscow, Russia (IBMP) [2, 21]. In those studies, it was also determined that performing pROM exercises with an intensity of 50% 1RM leads to the release of growth hormone, insulin-like growth factor, and cortisol into the blood. The results of this research and other literary data directly influence the rational design of the training process. It is necessary to the construction of a training session to understand that the use of pROM exercises during the training process can give both positive and negative results depending on the timing and placement within the overall training program. Therefore, it is important to develop correct methodological recommendations for exercise in order to induce specific physiological adaptation that stimulates performance enhancement without negatively interfering with the process of developing sport-specific skills.

Further research is needed to observe and elaborate possible implementation of pROM vs. fROM resistance training exercises over a longer period of time and the impact both may have on executing complex sports-specific skills such as the soccer penalty kick. Research supports that pROM exercises can be performed to develop muscular strength and hypertrophy at the end of a technical skill development training session, due to the accumulation of hydrogen ions caused by metabolic stress [32]. Some potential mechanisms and outcomes of interest are muscle time under tension and muscle activation [21]. Athletes and coaches could perform pROM resistance training exercise in order to induce specific metabolic stimulus promoting muscle strength and hypertrophy adaptations.

### 4.2. Limitation of This Study

The present study is limited by the facts that the sample comprised only youth players and a small number of participants; therefore, our findings cannot be extrapolated to athletes from other competitive levels or experience.

## 5. Conclusions

The novelty of this study is that pROM resistance training exercises should not be performed at the beginning of a sports-specific training session, since fatigued muscles will not allow effective performance of the precision motor actions during the development of sport-specific skills, such as a soccer kick, as demonstrated within this study.

Before performing sport-specific exercises and drills to develop technical skills, you should warm up for at least 15 minutes. The warm-up should include 5 minutes of slow running, 5 minutes of juggling with the ball, and 5 minutes of dynamic stretching of the major muscle groups to be worked during training. Next, fROM squats with a barbell can be performed utilizing a load that corresponds to 50% of the athlete's body weight for a duration of 40 seconds per set with a set tempo of 56 bpm; approximately 10-12 repetitions are performed during the 40 set. The use of fROM resistance training exercises after the warm-up may allow the athlete to potentiate the neuromuscular system for performing precision motor actions, such as a penalty and free kicks.

To assess the accuracy of the footballers' strikes, a special testing method was developed—highly qualified footballers performed a series of 25 strikes on the target (1 × 1 m square) from 11 meters and 15 strikes on a given half of the goal—from 30 meters. The metrological parameters of the methods for assessing the accuracy of strikes showed that the average accuracy of strikes into a meter target was 77%, the error—10%. The correlation between the first and second series was trr = 0.92.

Comparison of training mesocycles with the use of dynamic and static-dynamic strength exercises showed a higher efficiency of static-dynamic strength exercises for solving the problems of increasing the physical and technical readiness of young football players.

## Figures and Tables

**Table 1:** Statistical data of participants.

Type of football player participating	Football = 24 participants Indoor football = 12 participants
Experience	5-8 years
Position	Midfielder (4) and forward (2) in each subgroup of 6
Time with team	8 months or more
Age range	14.5 ± 0.5 years
Body weight range	56.3–69.5 kg
Height range	170 ± 5.9 cm
Gender	Only males were included in this study

**Table 2:** Average values of blood lactate and accuracy of penalty kicks before and after performing fROM exercises.

fROM					
Before			After		
	Accuracy	Lactate		Accuracy	Lactate
A	57.33 ± 12.56	2.98	A	48.0 ± 7.15	4.55
C	33.14 ± 8.8	3.11	C	37.71 ± 11.74	4.41
P	38.4 ± 14.5	1.98	P	38.4 ± 9.2	3.04
Total	42.96 ± 13.39	2.69 ± 0.2	Total	41.37 ± 17.25	4 ± 1

**Table 3:** Average values of blood lactate and accuracy of penalty kicks before and after performing pROM exercises.

pROM					
Before			After		
	Accuracy	Lactate		Accuracy	Lactate
A	62.66 ± 4.84	2.96	A	36.0 ± 5.65	10.75
C	36.8 ± 12.45	2.32	C	19.2 ± 5.21	11.28
P	36.8 ± 9.54	2.16	P	18.4 ± 11.52	8.84
Total	45.42 ± 14.93	2.48 ± 0.42	Total	24.53 ± 10.2	10.29 ± 1.3

## Data Availability

The data presented in this study are available on request from the corresponding author.
